# Use or Non-Use of Gerontechnology—A Qualitative Study

**DOI:** 10.3390/ijerph10104645

**Published:** 2013-09-30

**Authors:** Ke Chen, Alan Hoi-shou Chan

**Affiliations:** Department of Systems Engineering and Engineering Management, City University of Hong Kong, Hong Kong; E-Mail: kechen2-c@my.cityu.edu.hk

**Keywords:** attitudes, barriers, facilitators, gerontechnology, individual and group interviews

## Abstract

This study employed a qualitative approach to explore the attitudes and experiences of older people towards using gerontechnology, and to determine the underlying reasons that might account for their use and non-use of gerontechnology. Four focus group discussions and 26 individual interviews were undertaken. Qualitative data were analyzed using NVivo software and were categorized using coding and grounded theory techniques. The result indicated that old people in Hong Kong had an overall positive attitude toward technology. Positive attitudes were most frequently related to enhanced convenience and advanced features. Negative attitudes were most frequently associated with health risks and social problems arising from using technology (e.g., social isolation and addiction). Usage of technology is driven by outcome expectations and social influences, and supported by facilitators, whereas non-use of gerontechnology relates to the personal (e.g., health and functional capacities), technological (e.g., cost and complexity), and environmental barriers experienced. Use of gerontechnology is a synthesis of person, technology, and environment. To encourage non-users to adopt technology, there is a need to remove barriers at personal, technological, and environmental levels.

## 1. Introduction

The population of Hong Kong has experienced rapid aging in the past 20 years. The number of people aged over 60 exceeded 1.45 million in 2013, accounting for 20.1% of the total population, and this number is expected to increase to 3.37 million (42.1%) by 2050 [1]. As for the old-age dependency ratio in Hong Kong, ten persons of working age (*i.e.*, people aged 15 to 59) supported one dependent old person (*i.e.*, people aged at 60 and over) financially in the early 1980s, but the ratio has dropped to three persons supporting one dependent old person in 2012. The increase in the proportion of older population together with a shrinking working population would slow down the economic growth and result in a substantial rise in expenditures on healthcare and social welfare [2,3]. As well, there would be an increasing demand for old-age security, elderly services, patient beds, residential care homes, and community elderly care. In the Chinese tradition, family members are the main caregivers in the informal care of old people. The recent changes in the structure and lifestyles of Hong Kong family, including separate residences for adult children and elderly parents, reduced family size, women’s participation in the labor force, and balancing work and caregiving responsibilities, may hinder the practices and performance of traditional caregiving [3,4,5].

The emergence of gerontechnology has been shown to be a possible solution for the aging-related health, family and social burdens [6,7,8]. Gerontechnology combines gerontology and technology, and involves the research and development of techniques, technological products, services and environments based on knowledge of ageing processes [9]. Recently, research has shown that use of a variety of gerontechnology by older adults can help them to lead lives that are healthier, more independent, and more socially engaging on a continual basis. For instance, decline of physical and cognitive functions due to aging and sickness can be partially compensated for by assistive and health technologies; and consequently old people can stay longer in their own homes [6]. The development of telecommunication can remotely connect elderly patients with health professionals and care givers, and thereby to accomplish the shift from traditional institution-based health caring to home/community-based health care [7,8]. The Personal Emergency Link Service (PELS) developed in Hong Kong allows older people to gain access to timely emergency assistance by pressing an alarm button or by use of monitored wireless video cameras when staying at home or going out [10]. In addition, communication technologies like mobile phones, computers and the Internet enable older people to establish remote connections with family members and friends and go some way towards meeting the need for social interaction [11,12]. It is obvious that use of technology by seniors can not only help to satisfy their demands and requirements, but also has the potential to give support to caregivers and to reduce spiraling health care costs [13].

Although such technologies are supportive for daily life, older people do not show as much interest in adopting new technologies as the young, and for various reasons they are less likely to use technology [14]. According to the statistics released by the Hong Kong Census and Statistics Department [15], over 97% of 10–34 year-olds reported Internet usage, compared to only 15.5% of adults aged 65 and older. In the study of Olson *et al.* [16], compared with younger people, older adults were inclined to use old-fashioned technology rather than more recent technology in the domains of communication, customer service, health care activities, and home-based systems. These findings suggest that older people are still a disadvantaged group in terms of access to and usage of more recent technologies.

Factors influencing usage of technology have been studied extensively. The theoretical models employed to study technology usage behavior include the theory of reasoned action (TRA) [17], the technology acceptance model (TAM) [18], the technology acceptance model 2 (TAM2) [19], and the unified theory of acceptance and use of technology (UTAUT) [20]. From studies involving these models, a number of determinants have been found to impact on technology usage by older adults, including perceived usefulness [21,22], perceived ease of use [12,23], attitude towards technology [14,24], cognitive ability [25,26], social influences [22,27], self-efficacy [24,28], and facilitating conditions [27].

Most current studies have been conducted to test or confirm factors in the models of technology acceptance, and their findings have been based mainly on large-scale surveys, such as in the works of Braun [22], Pan and Jordan-Marsh [27], and Chung *et al.* [21]. Surveys can be useful when a researcher wants to identify statistical relationships between variables. However, this approach fails to gain understanding of underlying reasons and motivations contributing to technology usage. For instance, little is known about how older people appraise technologies. What are the barriers that older adults experience? Is there any difference between the reasons for use and reasons for non-use? In what ways could the use of technologies by older adults be encouraged? Previous explanations that address these questions in survey studies have been mainly based on assumptions and speculation rather than empirical investigation.

In addition, studies in the TAM field have mainly focused on the motivation to use technology, whereas, far too little attention has been paid to why older adults fail to use technology. The few studies that have attempted to explore the reasons for non-use of technology have produced rather contradictory findings. The study of Venkatesh and Brown [29] reported that non-computer users were mainly constrained by the barriers, while Melenhorst, Rogers and Bouwhuis [30] indicated the absence of advantage rather than barriers was the determinant for non-use of technology.

Unfortunately, most current studies concerning older people’s attitudes or usage of technologies have tended to focus on a limited number of technologies, particularly computers [11,31], and the Internet [32,33]. Less attention has been paid to other application domains such as housing, mobility, education, and recreation. A few recent studies have explored usage of several types of technology [34,35], however, there is still insufficient data regarding the use of a variety of different types of technologies by elderly people. 

No study so far has empirically explored how older adults define and appraise technology and why they decide to use or not use technology. Therefore, a thorough understanding of older people’s perceptions, experiences, and reasons for use and non-use relating to technology is needed. It is believed that more extensive qualitative studies can supplement the quantitative findings from previous studies, and explore in more depth the use and non-use of technology by older Hong Kong people.

## 2. Purpose of the Study

The purpose of the current study was to employ a qualitative approach to explore older people’s attitudes towards and experiences with using technology, and to determine the underlying reasons that might account for their use and non-use of technology. “*Gerontechnology*”, as a concept in this study, is interchangeable with “*technology*”, which refers to electronic or digital products or services that can increase independent living and social participation of older persons in good health, comfort, and safety. Specifically, this study was not limited to a specific technology but was concerned with a wide range of technologies in use in daily life. This study examined: (1) older adult attitudes toward using technology, (2) the reasons for use and not use of technologies, and (3) the personal and environmental facilitators influencing the use of technologies. The results of the study will be used to contribute to theory building that will hopefully lead to a better understanding of older adults’ technology usage.

## 3. Method and Procedures

### 3.1. Research Design

A triangulation of qualitative methods with a combination of individual face-to-face interviews and focus group discussions were adopted in this study. Qualitative studies are useful for obtaining better understanding of a phenomenon, because the methods allow and can encourage individuals to use their own words to express attitudes and experiences [36]. Several studies have revealed that focus groups are particularly useful for cataloguing the range of individual experiences, whereas individual interviews contribute to filling in the details for these experiences [37,38]. Accordingly, the focus groups method was adopted to capture a broad and diverse range of individual attitudes and thoughts concerning technology, and reasons for using or not using technology, as well as barriers and facilitators influencing older people’s usage of technology. Individual interviews were conducted to further explore personal usage experiences at a more detailed level. 

### 3.2. Procedure

Elderly service centres in Hong Kong were used as the sampling frame. Informed and written consent was obtained from all participants. Participants were informed they had the right to withdraw from the study at any time without giving a reason, and their information would be kept confidential and anonymous. They were required to fill in a questionnaire to supply information about demographics (age, education level, economic status compared with others in the same local area, general health conditions, *etc.*) and technology usage. At the beginning of each interview and group discussion, the interviewer gave a brief introduction and played a two-minute video called “*A Day in the Life—Technological Products and Services*” that documented a broad range of everyday technologies that elder individuals might use in a typical day. The video started with an electronic alarm clock at 6:00 a.m., followed by refrigerator, mobile phones, lifts, TVs, ATMs, and so on in different times in a day, and ended with a washing machine at 21:00 p.m. In the focus group discussion, participants were asked to introduce themselves and, as an “*ice breaker*” to name their favorite technology products with the reason for it being their favorite.

An interview guide was used by the interviewer to ensure that all principal questions were addressed in focus group discussions and individual interviews. The principal questions, for example, were: “*In your opinion, what is technology*?”,“*What technologies do you use in your daily life*?”,“*What do you like and dislike about technology*?”,“*What are the main reasons for using technology*?”,“*What are the main reasons for not using technology*?”,“*What are the difficulties/barriers when you are using technology*?”, *and* “*Are there any ways that can support technology usage by older adults*? *What are they*?”. Most questions were open-ended that started with “*how*” or “*what*” to produce detailed responses. Probing questions, such as “*please tell me more*” and “*please describe a real life situation*” were used to elicit individual experiences with using gerontechnology. The individual interviews and focus groups were conducted in Cantonese and lasted around 60 min, taking place in elderly centres. With permission of the interviewees, individual interviews and focus group discussions were audio recorded.

### 3.3. Participants

There were 50 participants all of whom were community-dwelling older adults (44 females and six males), ranging in age from 55 to 85 years (mean = 67.47; standard deviation = 7.96). The sample size of this study falls within the range of 15–60 suggested by Angrosino *et al.* [39] for qualitative studies. It is larger than the mean sample size (mean = 31) of 560 studies using qualitative interviews [40]. The sampling process continued until data saturation, or no new information or concepts are generated. The four focus groups consisted of 24 of the participants in total, and 26 respondents participated in individual interviews. Most participants had obtained primary education and above (64%), lived with family members (84%), and were of middle economic status compared with others in the same local area (86%). Fifty percent of the participants self-reported fair health conditions; 44% reported their health conditions to be excellent or good; only 6% reported poor or very poor health conditions. More detailed demographics are shown in Table 1. Background information about the participants’ use of technology is shown in Table 2.

## 4. Results and Discussions

### 4.1. Overview of Coding and Analyses of Interview Data

The audio taped data were transcribed verbatim and analyzed without revealing the identity of interviewees using NVivo 10 software package. A three-stage coding process was adopted here, including open, axial, and selective coding [39]. First, open coding was employed to categorize interview data and examine the consistency of transcripts. The frequently mentioned words or meaningful units were marked, extracted, and labeled with codes. Through constant comparison between transcripts, similar codes were combined into analytic concepts. Concepts were then grouped by similarity at a more abstract and theoretical level, whereby themes or categories were finally identified and the coding scheme were developed. In the present study, a total of 997 quotes were coded and categorized according to the coding scheme. 

The construction of the coding scheme combined the bottom-up/data-driven approach (grounded theory approach) whereby common concepts were extracted from a random number of transcripts, and the top-down/concept-driven approach whereby concepts were based on previous studies [12,29,34]. The coding scheme with proportions for each category/subcategory is shown in Table 3. Of the 997 quotes, 162 (16.25%) were coded as attitudes, 276 (27.68%) as reasons for use of gerontechnology, 388 (38.92%) as reasons for not using gerontechnology, and 171 (29.90%) as facilitators. 

**Table 1:** Demographics of the participants (*n* = 50).

Gender (*n*)	Age (mean)	Education (*n*)	Co-residence (*n*)	Marital status (*n*)	Means of living (*n*)	Economic status (*n*) ^1^	Health condition (*n*)
**Individual interviews (*n* = 26)**							
Men (5) Woman (21)	58–85 (65.92)	Informal (2) Pre-primary (3) Primary (8) Lower secondary (3) Upper secondary/Six-form (9) Post-secondary (1)	Family members (23) Alone (3)	Married (12) Divorced*/*Separated (8) Widowed (6)	Salary (1) Retirement wages (5) Property income (6) Family members (14)	Rich (5) General (21)	Excellent (6) Good (5) Fair (13) Poor (1) Very poor (1)
**Focus group 1 (*n* = 5)**							
Woman (5)	55–67 (59.20)	Pre-primary (1) Lower secondary (1) Upper secondary/Six-form (3)	Family members (5)	Married (1) Divorced*/*Separated (4)	Property income (1) Family members (4)	General (5)	Excellent (2) Good (3)
**Focus group 2 (*n* = 6)**							
Woman (6)	70–85 (78.83)	Informal (2) Pre-primary (2) Primary (2)	Family members (4); Alone (2)	Married (2) Widowed (4)	Family members (6)	General (6)	Excellent (1) Good (1) Fair (4)
**Focus group 3 (*n* = 8)**							
Men (1) Woman (7)	64–78 (71.88)	Informal (4) Pre-primary (3) Primary (1)	Family members (7) Alone (1)	Married (3) Divorced*/*Separated (2) Widowed (3)	Family members (8)	General (7) Poor (1)	Excellent (3) Fair (5)
**Focus group 4 (*n* = 5)**							
Woman (5)	55–78 (63.00)	Pre-primary (1) Primary (1) Lower secondary (2) Upper secondary/Six-form (1)	Family members (3) Alone (2)	Married (1) Divorced*/*Separated (1) Widowed (3)	Retirement wages (1) Property income (1) Family members (2) Local government (1)	General (4) Poor (1)	Excellent (1) Fair (3) Poor (1)
**Total (*n* = 50)**							
Men (6) Women (44)	55–85 (67.46)	Informal (8) Pre-primary (10) Primary (12) Lower secondary (6) Upper secondary/Six-form (13) Post-secondary (1)	Family members (42) Alone (8)	Married (19) Divorced*/*Separated (15) Widowed (16)	Salary (1) Retirement wages (6) Property income (8) Family members (34) Local government (1)	Rich (5) General (43) Poor (2)	Excellent (13) Good (9) Fair (25) Poor (2) Very poor (1)

^1^ Self-reported economic status compared with others in the same local area. The response choices include “very poor” (much lower than others), “poor” (lower than others), “general” (the same as others), “rich” (better than others), and “very rich” (much better than others).

**Table 2:** Background information about technology use.

Gerontechnology products or services	Usage rates
Induction cooker or microwave oven	92%
Remote control devices	94%
Emergency alert products/services	14%
Automatic teller machine (ATM)	68%
Credit cards	62%
Mobile phones	98%
E-mail	46%
Computers or Internet	70%
Transport smart cards	98%
Health massage products/sports equipment	78%
Electronic sphygmomanometer/glucometer	86%
Digital camera	72%
MP3/CD/DVD/VCD players	70%

**Table 3:** The coding scheme and proportion of the code (*n* = 997).

Categories (%)	Subcategories (%)	Definition	Codes (%)
Attitudes toward gerontechnology (16.25%)	Positive attitudes (56.17%)	A person’s favorable evaluation of the gerontechnology.	Convenience (46.15%)
			Social trend (17.58%)
			Advanced (13.19%)
			Speediness (12.09%)
			Unspecified (10.99%)
	Negative attitudes (43.83%)	A person’s unfavorable evaluation of the gerontechnology.	Health risk (19.72%)
			Addiction (18.31%)
			Older *vs.* younger (15.19%)
			Unsafe/lack of privacy (14.08%)
			Social isolation (11.27%)
			Frequent updates (8.45%)
			Environmental pollution (7.04%)
			Complex (5.63%)
Reasons for use (27.68%)	Utilitarian outcomes (63.04%)	The extent to which using a technology is perceived to be instrumental in achieving valued outcomes.	Convenience and speed (29.89%)
			Ease of communication (22.41%)
			Useful (18.39%)
			Knowledge acquisition (14.94%)
			Pass time (6.90%)
			Money saving (5.17%)
			Social interaction (2.30%)
	Hedonistic outcomes (14.49%)	The extent to which the individual is curious during the interaction and finds the interaction intrinsically enjoyable or interesting.	Interest and fun (75.00%)
			Like to learn (17.5%)
			Curiosity (7.5%)
	Social outcomes (6.16%)	The degree to which using a technology is perceived to enhance one’s image or status in one’s social system.	Image
	Social influence (16.30%)	The extent to which social values and members of a social network influence the usage behavior.	Fitting in with modern society/times
Reasons for non-use (38.92%)	Dispositional barriers (64.95%)	Personal factors associated with individuals’ attitudes and self-perceptions about oneself as a user.	Memory loss (21.03%)
			Lack of knowledge (16.27%)
			Anxiety (14.29%)
			Lack of necessity (9.92%)
			Difficulty with/inability to learn (9.52%)
			Do not know how to use (8.73%)
			Health and ability reasons (6.75%)
			Old age (3.57%)
			Perceptions of prejudice and discrimination (3.17%)
			Inconvenience others (3.17%)
			No interest (1.98%)
			Not smart/too dull (1.59%)
	Situational barriers (16.75%)	Personal factors which are beyond one’s control and are related to the individual’s life situation or environment at a particular time.	Lack of assistance (30.77%)
			Lack of time (27.69%)
			Limited exposure to modern technology (18.46%)
			Inaccessibility (16.92%)
			Secondary sources (6.15%)
	Technological barriers (18.30%)	Factors attributable to the features of gerontechnology product or service.	Expense (42.25%)
			Complexity (36.62%)
			Safe and privacy issue (21.13%)
Facilitators (17.15%)	External	Perception of internal or external factors that support technology use.	Training (27.49%)
			Assistance (25.15%)
			Adaptive design (14.62%)
			Encouragement (10.53%)
			Bought or given by family members (8.19%)
			Good tutor (5.26%)
	Internal		Positive attitudes to oneself (8.77%)

### 4.2. Range of Technologies Reported

Of the 997 coded quotes, 592 discussed technology in general, but the remaining 405 statements specified a certain type or form of technology. These technologies can be classified into five domains based on the similarities for their goals or context of use, viz. information and communication, home and daily living, entertainment and leisure, shopping and purchasing, and health care. The frequencies with which the technologies were discussed are shown in Table 4. The classification of gerontechnologies in the present study is different from those in the classic presentation of gerontechnology (see Bronswijk, Bouma, and Fozard [41]), five application domains include health and self-esteem, mobility and transport, housing and living, communication and governance, and work and leisure) in the following aspects: first, technology in this study was limited to digital or electronic devices; second, only one technological device for the purpose of transportation was mentioned, that is Octopus cards. Therefore, the mobility domain is combined with daily life domain. Third, all the participants were retired, therefore the work domain was eliminated. Fourth, participants expressed different attitudes and usage patterns with regards to technologies with or without money transaction. Therefore, the shopping and purchase domain was separated from daily living domain.

**Table 4:** The frequency with which technologies were discussed in each domain (*n* = 405).

Domains	Basic for domains	Frequency in domain	Technology	Frequency
Information and communication	Computer-based technologies and digital communication technology.	265	Computers	93
			Mobile phones	93
			Email	18
			Facebook	16
			Internet	16
			Smart phone instant messaging applications	14
			Tablet computers	11
			Video calls	4
Home and daily living	Support home and daily tasks.	60	Microwave ovens	25
			Octopus cards	12
			Induction cookers	11
			Rice cookers	9
			Wash machines	2
			Webcam for monitoring	1
Entertainment and leisure	Used on voluntary basis with free time.	36	Television	11
			Digital cameras	9
			Sports equipment	9
			DVD/VCD	7
Shopping and purchase	Use money	29	ATM	12
			Electronic-services (online transaction)	10
			Credit cards	7
Health care	Manage health	15	Emergency alarm services	11
			Sphygmomanometer/blood glucose meters	3
			Hearing aids	1

These data show that older adults are aware of and willing to use various types of technology, particularly computers and mobile phones. Surprisingly, health technologies were the least reported by the respondents. This result might have been due to the fact that 94% of the respondents reported they were generally healthy. Therefore, their immediate living needs may not be medical and health care related; rather, they were concerned about matters related to their lifestyles such as communication, social adaptation, home and living, leisure and entertainment, and shopping.

### 4.3. Definition of Technology by Participants

A concern for this study was how older people define technology, thus the questions like “*what is technology*?” *and* “*What technologies do you use in your daily life*?” were asked. The most frequent responses were “*the newest is technology*”, and “*technology is the advanced electrical appliance*”. Such responses indicated that technology was considered by respondents to be new, innovative, and advanced. A utilitarian perspective on technology was noticeable in some interviewees: “*I think technology is making use of tools*, *machines, and electricity to replace manpower, in order to meet our daily needs and make our lives more comfortable*”. Several interviewees explicitly stated “*technology is computers/mobile phones/microwave ovens*”.

### 4.4. Attitude towards Gerontechnology

To explore older adults’ attitude towards gerontechnology, questions like “*What do you think of the idea of using technology*?” and “*what do you like and dislike about technology*?” were asked. Attitude towards gerontechnology here was defined as a person’s favorable or unfavorable evaluation of gerontechnology; accordingly the responses regarding attitudes were separated into positive and negative subcategories. A wide range of attitudes were expressed by participants. Of the 162 attitude quotes, 91 (56.17%) were positive whereas 71 (43.83%) were negative, that is, more positive attitudes were expressed than negative attitudes when older people talked about technology.

Convenience was the factor contributed most to older adults’ positive perceptions about technology, accounting for 46.15% of the positive data. Participants indicated they like technology because it can make activities easier and faster, especially for cooking, communication, and administrative activities like financial transactions or transportation. In addition, technology was perceived by respondents as a progressive trend (17.58% of the positive data), and therefore an individual refusing to use technology was considered to be heading for obsolescence and to be out of touch with society. The technology features of advancement (13.19%) and speediness (12.09%) also contributed to the positive appraisals. 

Participants also expressed negative attitudes towards technology. The reasons for unfavorable attitudes were associated with health risks (19.72%), technology addiction (18.31%), generation gap (15.49%), unsafe (14.08%), social isolation (11.27%), rapid rate of change and updates (8.45%), environmental pollution (7.04%), and complex (5.63%). Respondents reported that they dislike using gerontechnology because these products are almost all electronic products which might produce radiation and cause adverse health effects. Likewise, using technological products, particularly computers, for a prolonged time could pose health risks including eyestrain, headaches, as well as muscle and joint pain. Moreover, some social problems arising from using technology, such as technology addiction, social isolation, and electronic waste were also reported as reasons for disliking technologies. Technology features—including rapid rate of change, need for updating, insecurity, and complexity—contributed to the negative attitudes as well. In addition, a generation gap regarding modern technology was noted by some respondents. It appeared to some participants that the younger and older generations differed in the purposes for using and the ways of using technology, and the younger generation was more comfortable with changes in technology.

### 4.5. Reasons for Use

A primary goal of this study was to understand why older people use or do not use technology products and services. The results indicated that reasons for adopting technology by respondents were mainly associated with the perceived consequences of using (83.69% of the reason-for-use data), which could be classified into utilitarian outcomes, hedonistic outcomes, and social outcomes. This finding corroborates those reported by Braun [22] and Mitzner *et al.* [34] in regard to older people’s motivation to use technology being determined by perceived benefits. Utilitarian outcomes were the most frequently mentioned reason for using technology, accounting for 63.04% of the reasons-for-use data. Respondents were motivated to use technology for reducing effort required and enhancing effectiveness in performing daily activities, which was described by participants as “*convenience and speediness*”. Considering that almost all of the participants were retired, using technology for utilitarian purposes was understandably concerned with personal activities rather that with work activities. The personal activities included household activities, learning, communication, leisure, social interaction, and health maintenance. Besides, they felt it necessary to use technology in order to cope with daily needs and problems. For instance, respondents with poor health reported that they needed health technology products or services to compensate for physical weakness.

Other instrumental outcomes related to using technology included knowledge acquisition, passing time, and maintaining connections with others. In addition, using technologies like various smart phone applications, free online services for books and movies, and Elder Octopus Cards (*i.e.*, transport smart cards for elders in Hong Kong) can help the elderly users save money. Some participants considered the use of communication technologies as a social activity that could widen social circles or help to meet friends. It is worth noting that trying to make connections with the younger generation was an important reason for adopting modern communication technology by older adults. One of the interviewees reported that her motivation to use the new Instant Messaging innovation was to “*build good relationship with younger children and fit into their lives*”.

Apart from the utilitarian outcomes, some participants attributed their usage to attaining favorable social outcomes, that is, enhancement of one’s image or status in social systems. For example, interviewees gave the following responses “*I start to use computers because I do not want to be labeled as outdated*”, and “*I feel more useful if I can learn to use some advanced technology*”.

Other than the external outcomes mentioned above, hedonistic reasons for use were also reported. Respondents indicated that they had a general curiosity about and became interested in new technology. Also, they found their interaction with technologies was intrinsically enjoyable or interesting, as described by some participants “*using a computer for me is playing a game*”.

It was found that social influences exerted a great impact on older people’s usage behavior, accounting for 16.30% of the data coded as reasons-for-use. Social influence, in contrast to social outcomes, is a perceived “*social pressure to perform a behavior*” [29]. Participants revealed they decided to use new technology because they “*wanted to fit in with modern society*” and “*did not want to be left behind*”. The “*significant others*”, including family members, friends, and peers in their social networks, played an important role in influencing older people’s usage behavior.

### 4.6. Reasons for Non-Use of Gerontechnology

The study investigated why some older adults fail to use technology, and the results suggested a variety of barriers inhibit their usage behavior. According to the qualitative responses, reasons for non-use of technology can be conceptualized into three types of barriers adapted from the framework suggested by Cross [42], viz., dispositional barriers (64.95%), situational barriers (16.75%), and technological barriers (18.30%). 

The dispositional barrier as described by Cross [42] related to the one’s beliefs, values, attitudes and perceptions of oneself as a user. Forgetfulness was identified by older adults as a major difficulty to use technology, accounting for 21.03% of dispositional barrier data. For instance, interviewees stated they failed to use ATM or electronic-services because they were “*unable to remember the ATM Personal Identification Number or any numerical password*”. Also, the respondents frequently mentioned they easily forgot things, had difficulty in retaining recently processed information, and needed more time to absorb new knowledge. The major barrier to memorizing things might be because fluid intelligence, on which learning new skills and knowledge depends, weakens with aging [43]. It was also found that a significant number of the participants indicated health and ability related reasons for non-use. They claimed their health conditions (vision and hearing) were too poor to use technology; or that using technology would be harmful to their physical health. Some participants attributed their non-use to a lack of prerequisite knowledge, including the capability to read and write Chinese or English, as well as lack of technological background knowledge. In particular, older Chinese people often did not have a good knowledge of the English alphabet, thus they had difficulty in doing tasks like using a keyboard. 

Anxious or emotional reactions were expressed by the interviewees when it came to using computers or money-related technological services such as ATM or credit cards. Many respondents explicitly mentioned that they were afraid to use computers, in particular when computers were not their own. They feared inadvertent deletion of important documents, damaging a computer, and making mistakes they cannot correct when using computers. Some respondents did not use gerontechnology because they perceived it to be unnecessary. For instance, many interviewees indicated that there is “*no need*” for them to adopt the personal emergency alarm service because they were in good health. Also, participants hesitated to use advanced technological devices because they felt that too much mental effort was involved to learn how to use such devices, often involving taking training classes, taking detailed notes, and continuously reviewing information and practicing. Beyond that, some respondents reject use of gerontechnology simply because of a lack of interest, especially when compared to other activities like doing sports.

Negative self-evaluated beliefs were found to inhibit usage behavior. Some respondents attributed their non-use to self-perceptions such as of “*too-old-to-use*”, “*not smart*”, or “*clumsy*”. Also, it was noticed that some participants faced an awkward predicament: on one hand, they indicated a need of assistance from others when having difficulties; but at the same time, they were afraid this behavior would cause inconvenience to the helper. Also, the image that they had of how other people might think of their technology usage was found to be important for the participants. It was found that there was generally a negative prejudice or some discrimination attached to the use of assistive health technology like emergency alarm services; and when asked why participants did not use such technologies, they responded “*I am not that old” and “I do not need stuff like that*”. Likewise, one male interviewee mentioned the “*authority*” of older persons: “*since older people were not as good as younger people regarding modern technology*. *Being lectured or taught by the younger ones would weaken the authority of us*...* I feel embarrassed when I need to seek advice from the younger*”.

In addition to dispositional barriers, an individual’s current circumstance or situation beyond their control was found to impact on usage behavior. In this study, the situational barriers included lack of assistance; lack of time; the limited exposure to modern technology; inaccessibility; and influences of secondary resources. Complaints about lack of assistance when encountering difficulties were widespread among participants. One interviewee stated: “*our children are busy with work*, *and they come back home late. When we have difficulties*, *it is impossible to seek assistance from them*”. Lack of time also was identified as a situational obstacle to gerontechnology usage. Participants, especially women, were busy with taking care of their grandchildren, doing housework, participating in volunteer or other activities, so that they did not have free time for learning and using some technologies.

Moreover, participants expressed the view that using innovative gerontechnology was extra difficult for those did not grow up with it. They indicated that they were born without modern technology like computers and mobile phones, which means that modern technology is foreign to the elderly and thus they have to learn a completely new language and skill late in their lives. The generation-related lack of earlier experience has also been reported by Docampo Rama *et al.* [44]. They described that older people experienced more difficulties than younger persons which may be because older adults did not have the opportunity to get skilled in their formative period (before the age of 25 years). Consequently, they may follow their existing or earlier habits and usage pattern, and have difficulty to learn new procedures. Lack of physical access needed for use was another reason for non-use. Participants commented that facilities like computers or DVD players were owned and used by family members, and sometimes they were not allowed to use them at home. Gerontechnology usage behavior was also found to be influenced by secondary sources of information from televisions, radios, and newspapers. If messages and information conveyed by the mass media about technology were negative, the participants would hesitate to adopt the technology. For example, one interviewee said “*I heard from the newspaper that a microwave oven would produce radiation when in operation*, *thus I use it less now*”.

Moreover, participants attributed their non-use to features of gerontechnology—expense, complexity, and safety or privacy issues. If the expense which comprises the price of the product or service, training or education fee, and maintenance cost, exceeded the acceptable range of old people, they would refuse to use the technology. One interviewee stated that “*the monthly charge for Mobile Emergency Service is HKD$200*, *that is too expensive for me. If the price goes down*, *I could consider adopting it*”. Such responses suggested that expense of using is a major concern when using gerontechnology by the participants. In many cases, participants only used simple basic functions and they did not want to try out more advanced complicated functions. For instance, several interviewees indicated that the feature-rich mobile phones were too complicated for them; they cannot memorize what functions the different buttons serve. Moreover, participants reported safety and privacy concerns when using technology. They worried about intrusion into personal privacy and of being cheated by others, specifically when using ATM or electronic-services.

This finding, that barriers were the main reasons for non-use is consistent with the study of Venkatesh and Brown [29], but do not support the research of Melenhorst, Rogers and Bouwhuis [30]. In the study of Melenhorst, Rogers and Bouwhuis [30], the absent benefit was found to be more predictive than barriers in determining non-use of new communication technology, specifically e-mail; but for non-use of traditional communication technology, both barriers and absent benefits were important. From this viewpoint, since the present study focused on a wide variety of technologies including both new and traditional ones, findings between this study and the research of Melenhorst, Rogers and Bouwhuis [30] are not contradictory. In addition, there are similarities between the barriers expressed by older adults in this study and those described by Lai [10]; Lee, Chen and Hewitt [33]; and Wagner, Hassanein and Head [12].

### 4.7. Facilitators of Using Gerontechnology

In the present study, participants were asked to think about the ways that could help to support their technology usage. Among various types of facilitators identified by the participants, training was the most frequently mentioned, accounting for 27.49% of the facilitator data. A majority of participants indicated that they could not acquire the skills and knowledge necessary to use computers unless they received formal training from social services centres. At the same time, the success of the learning was determined to a great extent by the quality of the tutors. The quality of “*patient*” and “*slow-paced instruction*” were highly valued by the interviewees. Apart from formal training, older people indicated that if they were required to use a new technology or learn to operate a new function that is totally unfamiliar to them, they must rely on other people to help them, rather than self-learning. One interviewee expressed, “*If I buy a new washing machine, there has to be someone who could teach me and demonstrate how to use different functions... I would not learn by myself*”. An unexpected finding was that the participants did not prefer being taught by their children. The unfavorable attitudes towards coaching by children were attributable to the younger ones being “*impatient*” and “*their demonstrations were too fast to follow*”.

Moreover, timely assistance was reported to be necessary to support usage of technology. It was found that although participants disliked being taught by their children, when they encountered technological difficulties, they would turn first to their children or professionals for help rather than refer to instruction manuals. Participants also indicated that most of the technological products they used were given to them or bought by their children. Furthermore, some participants reported that verbal praise or material rewards could encourage gerontechnology usage, and the encouragement may act as incentive for older adults. For instance, an interviewee who was also a volunteer for computer classes for seniors said “*When you teach elderly people or your parents to use technology*, *it would come to the end if you told them they are* ‘*stupid*’. *You need to coax them, treat them like children, and keep telling them* ‘*you can do it*’, *and* ‘*you are good*’”.

In addition, participants indicated “*a simple device with large font*, *learnable and easy to use is suitable for older people*”, which suggested an adaptive design would facilitate gerontechnology usage. Since aging is associated with changes in functional abilities such as impairment in vision and hearing as well as cognitive ability, adaptive design devices with larger size of front, loud sound, and that are easy to learn and use would make the devices more user-friendly for seniors.

Besides the environmental facilitators, it was also found internal variables, viz., positive attitudes to aging and self-confidence were conductive to interaction between older people and technology. The positive beliefs, such as “*never too old to learn*”, “*I am capable of...*”, “*sense of worth*”, and “*feeling of accomplishment*” would stimulate older adults to study and learn as well as provide confidence for them.

### 4.8. Development of a Grounded Theory

**Figure 1:**
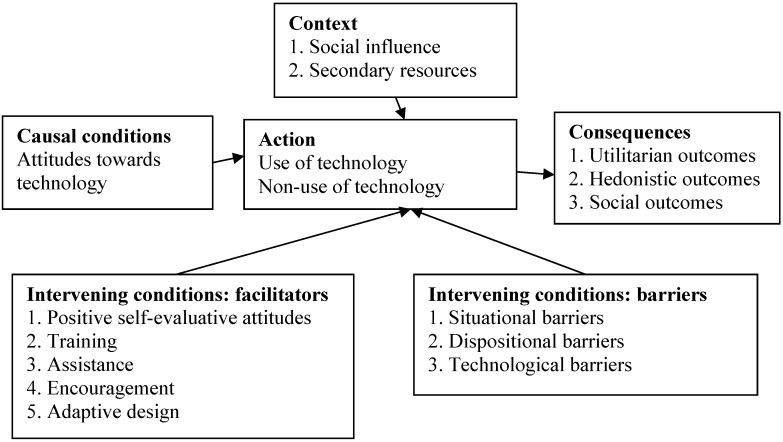
Result of the axial coding—relationships between categories.

The first stage of analysis identified concepts within categories. In the second stage of data analysis, axial coding was employed to link categories together and to explore their relationships. The process involved constructing a loose conceptual framework including causal conditions, context, action, intervening conditions, and consequences [39]. Result of the axial coding is illustrated in Figure 1. Since the reasons for use and non-use of technology were the main concern of this study, “*use or non-use*” was selected as the central action, which connected other categories and subcategories that divide into causal conditions, context, intervening conditions, and subsequent consequences.

**Figure 2:**
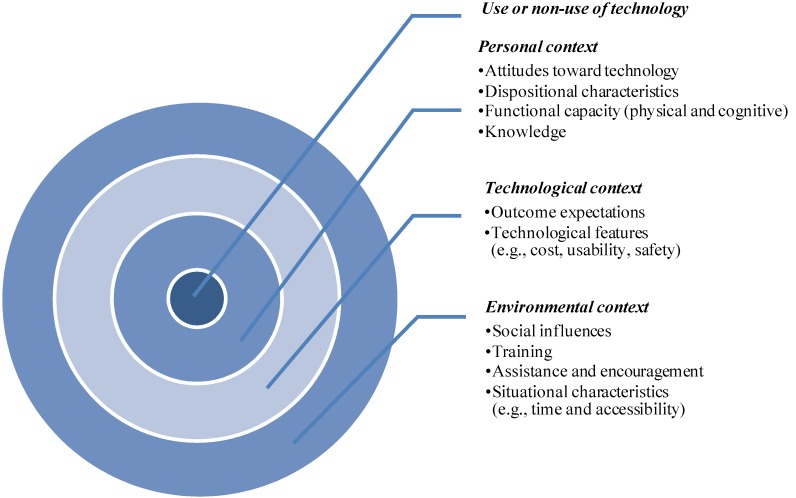
The grounded model based on qualitative data.

In the final selective-coding stage, all categories were re-examined and unified; and a resulting model, grounded in the qualitative data, was developed based on selective coding. Figure 2 shows the four-layer structure model. The grounded model suggested that the use or non-use of gerontechnology could be explained by the factors embedded in the personal, technological, and environmental contexts. In the model, each layer is influencing the core—use or non-use of technology—directly or indirect through the layer beneath it. 

The core or the inner layer of the grounded model is the use or non-use of technology, which is directly or indirectly determined by outer three layers. Older adults in the present study used a variety of technologies for communication, enhancing home and daily activities, shopping, entertainment, and managing health.

The second layer is the personal context which contains the characteristics and traits of the elderly users—attitudes to technology, health and functional capacity, dispositional characteristics, and knowledge and skills. The findings of this research are consistent with other studies [34,45], that have shown an overall positive attitude towards using technology. Also negative attitudes towards technology were found to be more varied than the positive attitudes. These results are in agreement with the study of Mitzner *et al.* [34], which found that older adults’ positive attitudes outnumbered negative attitudes towards technology in general, but negative attitudes were more varied.

The results here are also in agreement with findings of much of the previous work that has found associations between personal factors and usage of technology. The finding that older adults’ physical and functional capabilities are relating to technology usage has also been reported by previous researchers [31,32]. Also, consistent with previous studies [14,46], this study presents evidence that knowledge plays a key role in technology usage. 

The technological context is found in the third layer. Usage of gerontechnology was found to depend on the consequences of using and features of technologies. The result confirms the social cognitive theory suggestion that individuals are more likely to engage in behavior they expect will result in favorable outcomes [47]. Favorable outcomes cited by the elderly are associated with reduction in effort in performing daily activities (utilitarian outcome), image enhancement (social outcome), and perceived enjoyment (hedonistic outcome). These findings further support the motivation theory as an explanation for technology adoption [20,29]. The utilitarian and social outcomes serve as extrinsic motivators and using technology is to achieve instrumental outcomes, whereas hedonistic outcomes are intrinsic motivators derived from the pleasure and satisfaction associated with interaction with technology. In addition to the benefits of using, the specific features of gerontechnology of usability, expense, and safety are important in technology usage. Previous research has confirmed the importance of ease of use and found that due to age related physical and cognitive changes, older people are more likely to accept technologies that are easy to understand and have a simple interface design [32]. 

Besides direct influences, the variables in the technological context may also impact on usage through the mediation role of personal characteristics. For instance, in the TAM, technological features of ease of use and usefulness were antecedents of attitudes. Other research has reported that outcome expectations may affect attitude towards technology, and further influence behavior [25]. A further extension in the technological context is older people would intend to use if modern technology can be adapted to meet the specific needs of older individuals (like knowledge acquisition, social interaction, health care needs) as well as be designed to be flexible enough to be used by elderly with no limitations regarding to disabilities or circumstances.

The outer layer of the grounded model is environmental context, which comprised social influences, training and assistance, encouragement, and other situational characteristics. Older people in the study were found to be motivated to comply with prevailing values, norms, trends of a society, as well as important referents (especially family and circle of friends). Moreover, messages and signals conveyed through mass media, including newspapers, television, radio, and other secondary sources, also have an impact on an older individual’s behavior in this study.

In addition to the direct effect, previous research has provided evidence that social influences can indirectly impact behavior by altering an individual’s belief structure through internalization and identification [19]; or by mediation of personal attributes, such as self-efficacy and anxiety [47]. The study of Broady, Chan and Caputi [14] found that negative stereotyped views from tutors and peers may increase computer anxiety; however, other studies have reported that encouragement and perceived support from family and tutors can increase Internet self-efficacy and outcome expectations, which in turn may influence subsequent use or non-use behavior [11,48]. In addition, formal and informal training, e.g., demonstrations and instructions, and assistance from external parties facilitate usage among older people. Training programs or workshops may also help to build older adults’ confidence in using technology and lead to favorable attitudes, which may further increase usage intention [24,49]. These results agree with the finding from previous studies that facilitating conditions, including guidance and support from other people, financial resources, and accessibility, played an important role in determining attitudinal factors as well as technology usage [11,50].

Some results in the present study indicated that use of technology is embedded in a culture context. This can be illustrated briefly by the fact that limited knowledge of English was a barrier to those from a non-English-speaking background. Another finding was that participants expressed their dislike of being lectured to by a younger person, because that was perceived to be a weakening the authority of the older person. This may be due to the nature of Chinese culture which has been greatly influenced by Confucian values which place great importance on hierarchical status and authority. According to Confucianism, older people are superior to younger in terms of social and family status; and within a family, the older person is responsible for educating the younger, and the younger serves the older with filial piety and submission [51]. When it comes to technology usage, however, older people are at a disadvantage in comparison to the younger group. Consequently, being lectured to by younger people would pose a threat to an older person’s status or authority. This finding has not been reported by previous studies and might be accounted for by a culture-specific situation.

## 5. Conclusions

The present study employed a qualitative approach in order to achieve a clearer view of the attitudes of older adults towards technology, and to provide theoretical insight into the reasons for use and non-use of technology by older people in Hong Kong. The findings here indicate that usage of technology by older people is mainly driven by outcome expectations and social influences, and supported by facilitators, whereas, non-use relates to the personal, technological, and environmental barriers experienced. 

The grounded model based on the qualitative data indicates that use of technology is a synthesis of person, technology, and environment; there are some factors within the personal and situational contexts that have been overlooked in previous literature on TAM studies. Personal attributes, such as self-efficacy, physical and cognitive abilities, and knowledge were found to play important roles in shaping elderly individuals’ attitudes and behaviors. In addition, facilitating conditions including self-evaluative attitudes, training, assistance, and encouragement were found to have a significant impact on technology usage. Nevertheless, older people may not be involved with technologies for three reasons: first, their own personal circumstances such as lack of knowledge and functional impairment may impede their ability to use technologies; second, technological barriers like cost and complexity constrain their usage; third, the social environment including lack of assistance as well as limited exposure to modern technology impose restrictions on usage.

### 5.1. Implications

Results of this study provide some implications. This study suggests that older people’s technology usage behaviors are mainly motivated by perceived benefits, while barriers may restrain them from using technology. Consequently, for technological developers, effective design strategies might focus on ways to meet goals and expectations of older adults, maximize benefits like convenience and speediness, and provide supports for social and recreational activities. Likewise, some unfavorable features including concerns about safety, complexity, and health risks should be eliminated where possible or at least reduced to the point that the benefits of using the technology outweigh the costs.

It is evident from this study that training was the major factor facilitating technology use. Many previous studies have reported that attending training programmes or workshops can help older individuals to build self-confidence, elicit positive attitudes, and increase intention to use technology [24,47]. From a management perspective, it is suggested that training courses will increase the chance that older people will accept and utilize innovative technology. Further intervention should recognize the importance of training and education for enhancement of self-competence. In view of the fact that forgetfulness was the most frequently mentioned difficulty in using technology, tutors and helpers should give enough time for the elderly to learn and provide patient step-by-step demonstrations and guidance. Also, trainings should allow for the mental model of older adults and make use of their existing knowledge.

In addition, responses from the participants suggest that older adults did not like to be taught by their children. Similarly, the study of Xie [52] reported that Chinese older adults were more satisfied with technical support from *aged peers* who have experienced similar age-related changes and shared the same learning processes. Young people tend to be “*impatient*” because they do not seem to understand the learning speed, styles, and difficulties experienced by older adults. Therefore, training centres may consider recruiting senior tutors and volunteers because peer-coaching can provide more effective and efficient strategies to help seniors learn to use technology.

It is very important that a favorable learning and using environment should be created for older users because of the fact that social influences play an important role in affecting the behavior of older adults. Also, older adults placed a high value on the images that they had in their social system; the way elderly people are viewed and treated will greatly impact their usage of technology. This indicates that to promote technology use by seniors, it is important to avoid labeling the older person as “*technophobic*”. Media and public opinion also should promote the belief that seniors have the right, need, and capability to use technology. Relevant organizations and the community in general should create an atmosphere that is supportive and favorable to the raising of confidence of older people and encourages them to interact with technology. Young family members should encourage and assist older people to use and practice with gerontechnology products at home, and provide hands-on guidance and help for older users. 

### 5.2. Limitations

Although the present work investigated older people’s attitudes and usage of technology, the results are based on cross-sectional studies which did not take into account user experiences. Previous studies have found differences in the pre- and post-usage attitudes between users [47,53]. For example, Venkatesh and Davis [19] provided evidence that the impact of social influences on intention to use technology were strong during the early stages, but will weaken over time as experience is gained. Consequently, future work is necessary to understand more thoroughly the entire technology adoption life cycle, and longitudinal studies should be employed.

Technology usage by older adults is a multi-disciplinary topic, and it was found to be influenced by a variety of factors, involving personal characteristics, technological factors, and environmental variables. The grounded model here proposed reciprocal relationships between those factors and different contexts; however, how they are interrelated has not been fully studied. For example, the impacts of attitudes to using and self-evaluative beliefs on usage behavior have been studied, but could a person’s usage behavior also influence his or her attitudes towards using and self-evaluative beliefs? Further research might focus on exploring the reciprocal relationships between factors within different contexts. Also, it is suggested that usage of technology is embedded in the context of culture. Few studies have empirically examined cultural issues associated with technology usage. Future research may focus on identifying the major cultural dimensions and their corresponding relationships with technology usage by users.
